# Effective Treatment of Chronic Proliferative Cholangitis by Local Gentamicin Infusion in Rabbits

**DOI:** 10.1155/2018/6751952

**Published:** 2018-07-24

**Authors:** Qin Yang, Zhenru Wu, Fei Liu, Junke Wang, Wenjie Ma, Haijie Hu, Fuyu Li, Qiuwei Pan

**Affiliations:** ^1^Department of Hepatobiliary Surgery, West China Hospital of Sichuan University, Chengdu 610041, Sichuan Province, China; ^2^Department of Gastroenterology and Hepatology, Erasmus MC-University Medical Center and Postgraduate School Molecular Medicine, Rotterdam, Netherlands; ^3^Laboratory of Pathology, Key Laboratory of Transplant Engineering and Immunology, NHFPC; West China Hospital, Sichuan University, Chengdu 610041, China

## Abstract

**Background:**

Hepatolithiasis is highly prevalent in East Asia characterized by the presence of gallstones in the biliary ducts of the liver. Surgical resection is the potentially curative treatment but bears a high risk of stone recurrence and biliary restenosis. This is closely related to the universal presence of chronic proliferative cholangitis (CPC) in the majority of patients. Recent evidence has indicated the association of bacterial infection with the development of CPC in hepatolithiasis. Thus, this study aims to investigate the feasibility and efficacy of local infusion of gentamicin (an antibiotic) for the treatment of CPC in a rabbit model.

**Methods:**

The rabbit CPC model was established based on previously published protocols. Bile duct samples were collected from gentamicin-treated or control animals for pathological and molecular characterization.

**Results:**

Histologically, the hyperplasia of biliary epithelium and submucosal glands were inhibited and the thickness of the bile duct wall was significantly decreased after gentamicin therapy. Consistently, the percentage of proliferating cells marked by ki67 was significantly reduced by the treatment. More importantly, this treatment inhibited interleukin 2 production, an essential inflammatory cytokine, and the enzyme activity of endogenous *β*-Glucuronidase, a key factor in the formation of bile pigment.

**Conclusions:**

Local gentamicin infusion effectively inhibits the inflammation, cell proliferation, and lithogenesis in a rabbit model of CPC. This approach represents a potential treatment for CPC and thus prevents recurrent hepatolithiasis.

## 1. Introduction

Hepatolithiasis, the presence of gallstones in the biliary ducts of the liver, is a common disease in Asia with incidence rates ranging from 2% to 25% [1, 2]. It is recognized as an intractable disease because of the high reoperation rates and a well-known etiology of cholangiocarcinoma [3–5]. However, the exact etiology of hepatolithiasis is not clear [6].

Current therapeutic strategies for hepatolithiasis are surgical and nonsurgical treatments. Hepatectomy has been considered the potentially curative treatment for some cases, because it can eradicate the stones and the associated pathologic changes, including biliary stricture, fibrosis, and microabscess. However, it is more refractory to surgical treatment than most other benign diseases of the biliary tract. On the other hand, 42% to 75% biliary stricture rates were found in hepatolithiasis patients who underwent hepatectomy [7–12]. In these regards, hepatolithiasis is hardly curable and its long-term outcome is far from satisfactory.

Chronic proliferative cholangitis (CPC) plays an important role in the pathogenesis of stone recurrence and stricture information. A previous study has reported that bacteria are present in the bile of all hepatolithiasis patients and Gram-negative bacteria are the most abundant [13]. Thus, bacterial infection is thought to be the main cause of CPC. However, CPC in hepatolithiasis is currently persistent and refractory to routine antibiotics because of the abnormal organizational structure in the bile duct wall. Briefly, under the influence of infiltration of inflammatory cells and proliferation of fibrous connective tissue, the peribiliary glands are usually enclosed to form intricate branches and the blood vessels around these glands are stenosed or obstructed [14–16].

Gentamicin has been demonstrated to be very sensitive for most Gram-negative bacteria. It has been applied for treating wound infection, acute conjunctivitis, and urinary tract infection by local infusion [17–22]. Thus, we hypothesize that gentamicin might be effective in treating CPC via biliary infusion. Because medication through biliary infusion could directly function on the bile duct wall avoiding systematic side effects. Furthermore, antibiotics could remove the bacteria in the submucosa through penetration, regardless of the stenosis or obstruction of the blood vessels around the peribiliary glands. In these regards, we intended to investigate the feasibility and effects of local gentamicin infusion on CPC in rabbit models, aiming at developing a novel approach to treat CPC to prevent recurrent hepatolithiasis.

## 2. Materials and Methods

### 2.1. Animals and Study Design

Thirty-eight New Zealand white rabbits weighing 2-3 kg were purchased from the Experimental Animal Center of Sichuan University and were randomly divided into three groups: proliferative cholangitis group (PC group,* n*=16); antibiotic therapy group (AT, n=16); sham-operated group (SO group,* n*=6). The animal model of CPC was generated for PC and AT groups as previously described [23, 24]. Briefly, the rabbit's duodenal wall was punctured with a 26-gauge needle and then a 5-0 nylon thread was inserted into the common bile duct through the duodenal papilla. After the other side of the thread was fixed at the duodenal wall with a purse-string suture, it was left in the bile duct for 6 weeks. Then, a polyethylene tube was inserted into the common bile duct via an incision (about 1 mm). The tube was fixed on the common bile duct wall and 5 ml of saline solution was injected into the tube to prove no leakage. Finally, the other side of the tube was pulled out through the abdominal wall and fixed. Biliary infusion with 20 ml gentamicin solution (80,000 units) was performed (once daily) during the 6 weeks following surgery in the AT group. For the PC group, 20 ml normal saline was a replacement of antibiotic. For the SO group, only surgical dissection of common bile duct was performed. After six weeks, all the rabbits were sacrificed and the common bile ducts and stones in the ducts were collected for further analysis.

### 2.2. Histological Examination

Part of the collected bile duct sample of each rabbit was fixed in formalin (10% neutral buffered). Four-micrometer sections from paraffin-embedded tissues were stained with hematoxylin-eosin (HE). The thickness of the bile duct was measured at four points (two diagonal lines meeting at a 90 angle) along the bile duct and the mean value was calculated.

### 2.3. Immunohistochemistry

The tissue sections were incubated with the anti-ki67 primary antibody (Abcam plc Co., USA) overnight at 4°C, followed by incubation with the biotinylated secondary antibody for 1 hour at 37°C. The labeling index (LI) values of Ki-67 were evaluated by digital image analysis.

### 2.4. Western Blot

The Cox-2 protein expression in the common bile duct wall was detected by western blot as previously described [25] and semiquantitative analysis was performed by Image-Pro plus 6.0.

### 2.5. Enzyme Assay

Endogenous and exogenous *β*-glucuronidase (*β*-G) activity and concentrations of IL-2 in the bile duct wall were detected by the *β*-glucuronidase activity assay kit (BioVision, USA) and IL-2 rabbit enzyme-linked immunosorbent assay (ELISA) kit (Jiyinmei Co, Wuhan, China), respectively, according to the manufacturers' instructions. The same method was used to detect the exogenous *β*-G activity in the stones of common bile ducts.

### 2.6. Statistical Analysis

All data were analyzed using the SPSS version 19.0 software. Statistical significance was determined by one-way ANOVA and P value of less than 0.05 is considered as statistically significant.

## 3. Results

### 3.1. Local Gentamicin Infusion Decreases the Thickness, Hyperplasia Degree of Epithelium, and Submucosal Glands of the Bile Duct Wall

**Figure 1:**
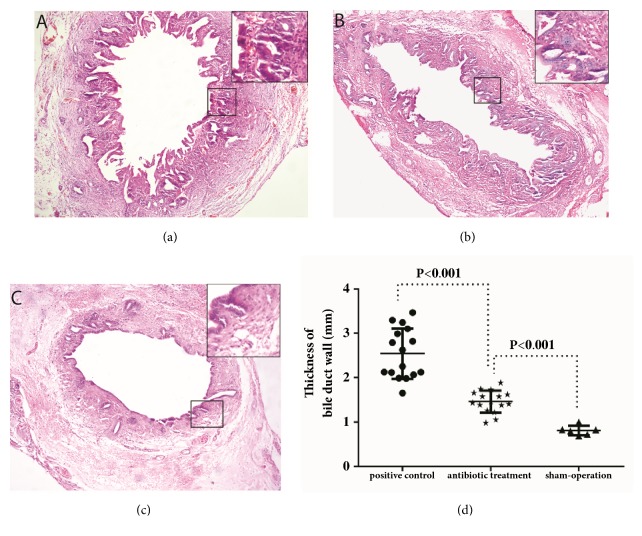
**Pathological changes of the common bile duct wall**. Hematoxylin-eosin (HE) staining of the common bile duct (original magnification ×50) showed the hyperplasia of epithelium and submucosal glands of the bile duct wall in positive control group (a) and the hyperplasia was inhibited by gentamicin in antibiotic treatment group (b), compared with those in sham-operation group (c). The thickness of the bile duct wall was measured (d) and a significant difference was shown between positive control and antibiotic treatment group. (Positive control versus antibiotic treatment group; antibiotic treatment vs. sham-operation group, p<0.001.)

In the rabbit model, CPC was induced as papillary hyperplasia in the biliary mucosa, inflammatory cell infiltration in the bile duct, submucosal glands hyperplasia, and fibrous thickening in the biliary duct wall. To evaluate the effects of gentamicin on cholangitis, we examined the pathological changes in the bile ducts through HE staining. In the samples analyzed, compared with the PC group (Figure 1(a)), the hyperplasia degree of epithelium and submucosal glands of the bile duct wall decreased obviously after gentamicin treatment (Figure 1(b)). Therefore, we measured the thickness of the bile duct wall by digital image processing. The results showed that the thickness of the bile duct wall in the AT group is less than half of that in the PC group (P<0.001), though there was still a significant difference between gentamicin treatment (AT group) and normal values (SO group) (p<0.001) (Figure 1(d)).

### 3.2. Inhibition of Cell Proliferation in the Bile Duct by Gentamicin Treatment

**Figure 2:**
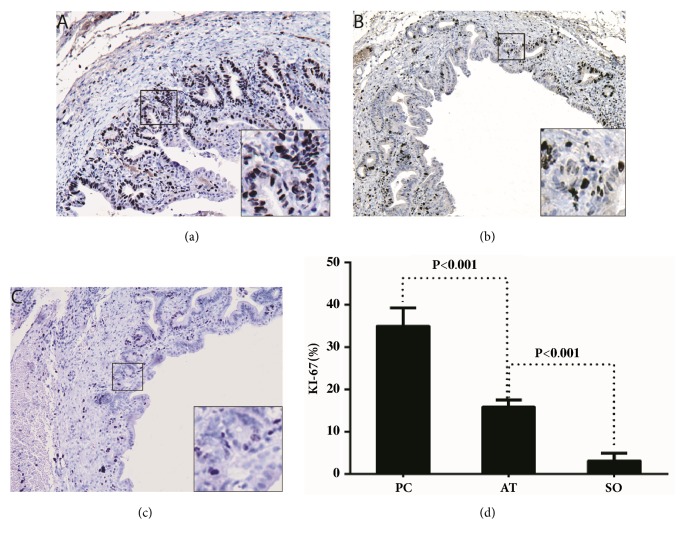
**Ki67 expression of the common bile duct wall (original magnification ×100)**. The obvious increase of ki67 expression was observed in positive control group (a) and only half of that level was observed after gentamicin treatment in the antibiotic treatment group (b) (antibiotic treatment versus positive control group, P<0.001), which both significantly higher than that in sham-operation group (c). And the ki67 labeling index (LI) of three groups (d) quantified the difference of ki67 expression among them (about 35%, 15%, and 5%, respectively).

Subsequently, we examined the expression level of ki-67 which is a widely used a marker for cell proliferation by immunohistochemistry. A high percentage of ki67 expressing cells were observed in the PC group (Figure 2(a)), which was greatly inhibited by gentamicin treatment (Figure 2(b)). The data of ki-67 labeling index (LI) (about 35%, 15%, and 5%, respectively) demonstrated that the expression level in AT group was significantly lower than that in the PC group (P<0.001) though it was still higher than that in the SO group (P<0.001) (Figures 2(c)–2(d)).

### 3.3. Gentamicin Inhibited the Inflammatory and Lithogenic Effects in CPC

**Figure 3:**
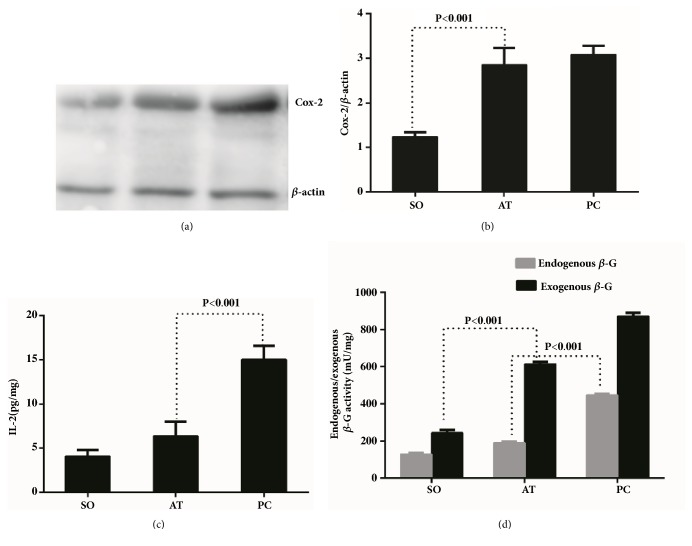
**Changes in inflammatory factors in the common bile duct wall and stones**. Cox-2 protein expression in the AT group was slightly decreased by gentamicin treatment comparing with the positive control group (a), but still significantly higher than that in the sham-operation group after semiquantified assessment (b) (sham-operation versus antibiotic treatment group, p<0.001). The concentration of IL-2 in the bile duct in the antibiotic treatment group was inhibited by gentamicin to almost normal (sham-operation versus antibiotic treatment group, p>0.05), which was significantly lower than that in the positive control group (antibiotic treatment versus positive control group, p<0.001) (c). The same tendency was observed in the endogenous beta-glucuronidase activity (d).

Both Cox-2 and interleukin 2 (IL-2) play crucial roles in the process of inflammation [26, 27]. The expression of the Cox-2 protein, examined by western blot, was only slightly inhibited by gentamicin without significant difference between the AT and PC group (Figures 3(a)-3(b)). In contrast, the IL-2 level in the AT group was significantly decreased compared with that in the PC group (P<0.001), although slightly higher than that in the SO group (P>0.05) (Figure 3(c)). Beta-Glucuronidase activity was known as the key factor in the formation of bile pigment. We detected the *β*-G activity of bile duct wall and stones by enzyme colorimetric assay to verify the inhibition of gentamicin on lithogenic potentiality. A significant decrease of the endogenous*β*-G activity was observed after gentamicin treatment (AT versus PC group, P<0.001; AT vs. SO group, P>0.05). However, this inhibitory effect was less potent in respect to the exogenous*β*-G activity (Figure 3(d)).

## 4. Discussion

Hepatolithiasis is recognized as an intractable disease because of the high incidence of stone recurrence and biliary stricture after surgical resection. However, its etiology is not clear and there is no effective management to prevent the recurrence. Targeting CPC in hepatolithiasis has been recently realized as a novel approach. The intrahepatic stones often injure the biliary mucosa and result in bacterial infection in the submucosal layer, as bacteria are present in the bile of all hepatolithiasis patients. This is the main cause of developing CPC, characterized by the hyperplasia of mucosa and submucosal glands and the proliferation of the fibrous connective tissue. The hyperplastic peribiliary glands and blood vessels around these glands are usually enclosed or obstructed by the condensed fibrous connect tissue to form intricate branches. Thus, bacteria could hide in these branches and cause new biliary infection which leads to repeated and persistent CPC [1, 16, 28]. More importantly, the proliferation of fibrous connective tissue in the bile duct wall could narrow the biliary lumen to form bile duct strictures, which would slow the bile flow and lead to bile stasis. Afterwards, bile sludge could fill in the bile duct and form new pigment stones to aggravate the hepatolithiasis or cause stone recurrence after choledochoscopic lithotomy, that is, a vicious cycle between hepatolithiasis and CPC.

Considering this pathogenic feature, we hypothesize that inhibition of CPC will be effective in preventing the stone recurrence and biliary stricture of hepatolithiasis after choledochoscopic lithotomy. Previous studies have investigated the antiproliferative effects of cytostatic drugs on CPC and some inhibition of the hyperplasia of the biliary epithelium, submucosal glands, and collagen fiber in the bile duct wall has been demonstrated. However, the cytotoxicity of these drugs was evident, thus limiting the clinical application [29–32]. As bacterial infection plays a key role, we have explored antibiotic treatment. In particular, targeting the Gram-negative bacteria is attractive, as they are abundantly present in the bile of hepatolithiasis patients [13].

However, CPC is resistant to routine antibiotics because of the abnormal structure of the bile duct wall. Classically, antibiotics reach the bacteria via blood circulation, but it is inefficient to reach the biliary submucosa and glands because the blood vessels around these glands are often stenosed or obstructed in these patients. In these regards, we believe that local antibiotics infusion, reaching the bacteria via penetrating the tissue, shall be an effective management for CPC. Local treatment of gentamicin has been applied for wound infection, acute conjunctivitis, and urinary tract stricture in the clinic because of its high tissue penetration and antibiotic spectrum and being sensitive to most Gram-negative bacteria. In this study, we applied local gentamicin infusion in CPC rabbit models and our data showed that gentamicin effectively inhibited the hyperplasia of the biliary epithelium, submucosal glands, and collagen fibers in the bile duct wall. Concurrently, the levels of inflammatory factors and the endogenous beta-glucuronidase activity, which is key to pigment stone formation, were decreased by local gentamicin treatment. Since gentamicin could be injected via the biliary drainage tube placed after choledochoscopic lithotomy, we propose that this approach is highly feasible to be implemented in the clinic to prevent the incidence of reoperation in hepatolithiasis patients.

In summary, our study has demonstrated the feasibility and efficacy of local gentamicin infusion for treating CPC in rabbits. Further studies are warranted to develop into clinical practice.

## Data Availability

The authors confirm that the included data supporting the findings were generated for the study. The data that support the findings of this study are available from the corresponding author, Fuyu Li, upon reasonable request.
