# Corrigendum to “Effective Treatment of Chronic Proliferative Cholangitis by Local Gentamicin Infusion in Rabbits”

**DOI:** 10.1155/2018/8305927

**Published:** 2018-11-04

**Authors:** Qin Yang, Zhenru Wu, Fei Liu, Junke Wang, Wenjie Ma, Haijie Hu, Fuyu Li, Qiuwei Pan

**Affiliations:** ^1^Department of Hepatobiliary Surgery, West China Hospital of Sichuan University, Chengdu 610041, Sichuan Province, China; ^2^Department of Gastroenterology and Hepatology, Erasmus MC-University Medical Center and Postgraduate School Molecular Medicine, Rotterdam, Netherlands; ^3^Laboratory of Pathology, Key Laboratory of Transplant Engineering and Immunology, NHFPC; West China Hospital, Sichuan University, Chengdu 610041, China

In the article titled “Effective Treatment of Chronic Proliferative Cholangitis by Local Gentamicin Infusion in Rabbits” [1], there was an error in the Acknowledgments section, which should be corrected as follows:

“This research was supported by Applied Basic Research Project of Sichuan Province (2018JY0019) and the China Scholarship Council for funding Ph.D. fellowships to Qin Yang (201706240113). The authors gratefully thank Professor Y. Shi for discussing this project.”
